# Long-term results of induction chemotherapy for non-operable esophageal squamous cell carcinoma followed by concurrent chemoradiotherapy: a single-centre experience

**DOI:** 10.2478/raon-2024-0038

**Published:** 2024-09-15

**Authors:** Geng Xiang, Guangjin Chai, Bo Lyu, Zhaohui Li, Yutian Yin, Bin Wang, Yanglin Pan, Mei Shi, Lina Zhao

**Affiliations:** Department of Radiation Oncology, Xijing Hospital, Air Force Medical University, Xi'an, Shaanxi, China; State Key Laboratory of Cancer Biology, National Clinical Research Center for Digestive Diseases and Xijing Hospital of Digestive Diseases, Xijing Hospital, Air Force Medical University, Xi'an, Shaanxi, China

**Keywords:** esophageal squamous cell carcinoma, induction chemotherapy, concurrent chemoradiotherapy, responder, propensity score matched

## Abstract

**Background:**

This study aimed to investigate the long-term clinical outcomes and toxicities of induction chemotherapy (IC) followed by concurrent chemoradiotherapy (CCRT) *vs*. CCRT alone in patients with non-operable esophageal squamous cell carcinoma (ESCC).

**Patients and methods:**

Between 2008 and 2022, 271 ESCC patients who received definitive CCRT based on intensity modulated radiation therapy (IMRT)/volumetric modulated arc therapy (VMAT) were enrolled. Through a propensity score-matched (PSM) method, 71 patients receiving IC and CCRT were matched 1:1 to patients who received CCRT alone. The Kaplan-Meier method and Cox proportional hazards model were applied to analyze survival and prognosis.

**Results:**

The IC + CCRT group had no improvement in 5-year overall survival (OS) rate, recurrence-free survival (RFS) rate, and distant metastasis-free survival (DMFS) rate (all p > 0.05) compared with the CCRT group. The 5-year OS rate (65.6% *vs.* 17.6% *vs.* 29.3%, p < 0.001), RFS rate (65.6% *vs.* 17.6% *vs.* 26.9%, p < 0.001), and DMFS rate (62.5% *vs.* 10.3% *vs.* 27.2%, p < 0.001) of the IC good responders were significantly higher than that of the IC poor responders and CCRT group. Multivariate analysis revealed that total radiotherapy time (≥ 49 days) and stage III/IV were independent predictive factors of OS, RFS, and DMFS. No significant differences were observed in the rates of grade 3–4 toxicities between both groups.

**Conclusions:**

Our results showed the addition of IC to CCRT was not superior to CCRT in unselected ESCC patients, while IC responders could benefit from this regime without an increase in toxicities.

## Introduction

Esophageal cancer (EC) is the sixth most common cause of cancer death worldwide, with more than 550,000 new cases of esophageal carcinoma diagnosed each year.^1,2^ Unlike most Western countries, esophageal squamous cell carcinoma (ESCC) is still the main pathological type in China.^3^ Regardless of its histological type, the overall survival (OS) of patients with EC is still poor.^4^ For the management of EC which is deemed a medically unresectable tumor, definitive concurrent chemoradiotherapy (CCRT) is the standard therapy, guaranteeing organ preservation and providing a better quality of life.^5^ However, although definitive CCRT results in encouraging short-term outcomes in the majority of patients, the prognosis remains unfavourable, with 5-year overall survival rates of 20%.^6^ Especially, the rates of locoregional recurrence (LR) and distant metastasis after definitive CCRT can be as high as 50%.^7,8^ Therefore, improvement in treatment intensity is greatly needed.

Induction chemotherapy (IC) is an attractive approach but also controversial. Theoretically, the additional IC followed by concurrent chemoradiotherapy (IC-CCRT) has potential benefits for early eradication of micro-metastases, increased tumor radiosensitivity, prevention of tumor progression, and even prolonged OS.^9^ Previous studies have suggested that IC-CCRT has better failure-free survival, overall survival, and distant failure-free survival than CCRT alone in nasopharyngeal cancer, and has been cited by National Comprehensive Cancer Network (NCCN) clinical guidelines.^10^ However, the addition of IC to CCRT in the management of ESCC is less reported, and the results of the retrospective studies showed conflicting results.^11,12,13^ A randomized controlled trial showed that compared to CCRT alone, the addition of induction chemotherapy with docetaxel plus cisplatin failed to significantly improve the response rate or survival outcomes in unselected ESCC, which were limited by staging and response evaluation issues.^9^ Previous research revealed that IC before CCRT was associated with improvements in pathological complete response (pCR) rate and survival in neoadjuvant therapy settings.^14,15^ Therefore, it is important to identify patients who may benefit from IC, especially the patients who are responsive to chemotherapy. Furthermore, the radiotherapy techniques in previous studies were mainly based on 3D-CRT or IMRT.^9,11,12,13^ The true value of IC for ESCC patients remains unclear in the era of modern technique IMRT/volumetric modulated arc therapy (VMAT), which could greatly improve the accuracy of radiotherapy with lower toxicity.^16^ To the best of our knowledge, there are no published researches to date that compared IC + CCRT *vs.* CCRT alone for the ESCC patients receiving IMRT/VMAT only.

Therefore, we performed this retrospective study to evaluate the long-term survival outcomes among the ESCC patients who were treated with IC + CCRT to better understand the feasibility, efficacy, and safety of this approach by propensity score matched (PSM) methods. We further performed a stratified analysis to analyze the relationship between tumor response to IC and treatment outcomes.

## Patients and methods

### Study population

We retrospectively reviewed data derived from patients with diagnosed ESCC between April 2008 and March 2022. All eligible patients met the following criteria: 1) considering non-operable or refusing surgery; 2) histopathological proof of ESCC (T1–4N0–3) without distant metastasis; 3) 18–70 years of age; 4) Eastern Cooperative Oncology Group performance status of 3 or below; 5) receiving either IC + CCRT or CCRT based on IMRT/VMAT; 6) adequate liver and renal functions; 7) either TPF (docetaxel + cisplatin + fluorouracil), PF (cisplatin + fluorouracil), or TP (docetaxel/paclitaxel + cisplatin) as the IC regime. 8) A radiotherapy (RT) dose of more than 50.0 Gy was defined as definitive. Additional information, including gender, pathological diagnosis, tumor location, date of diagnosis, chemotherapy pattern and drugs, radiation technology, and dosage were collected from the hospital outpatient follow-up database. This study was approved by the Ethics Committee of the First Affiliated Hospital of Air Force Medical University (ethical approval number: KY20172035-3).

### Treatment

#### Induction chemotherapy (IC)

For the IC + CCRT group, patients were given one to four cycles of IC based on doctor's choice. IC regimens consisted of TPF (docetaxel 60 mg/m2/day on day 1, cisplatin 50 mg/m2/day on days 1 to 2, and 5-fluorouracil 500 mg/m2/day on days 1 to 3), TP (docetaxel 60 mg/m2/day on day 1 or paclitaxel 150 mg/m2/day on day 1 and day 8, cisplatin 50 mg/m2/day on days 1 to 2), PF (cisplatin 50 mg/m2/day on days 1 to 2 and 5-fluorouracil 500 mg/m2/day on days 1 to 3). The cycles were administered every 3 weeks. Patients were treated with definitive CCRT within 3 to 6 weeks after the end of the last IC cycle.

#### Concurrent chemoradiotherapy (CCRT)

RT was given using IMRT/VMAT on the first day of chemotherapy in both groups as previously reported.^17^ All patients were fixed by thermoplastic body film. Briefly, the gross tumor volume (GTV) was defined as the primary tumor and lymph nodes considered positive by computed tomography (CT) and/or positron emission tomography/computed tomography (PET/CT), and endoscopic findings. The clinical target volume (CTV) was defined as the GTV plus an additional 3 cm craniocaudal expansion along the esophagus, and a 0.5 cm lateral margin. For tumors of the cervical or upper thoracic esophagus, the lymph nodes of the supraclavicular fossa were included in the CTV at the discretion of the physician. The planning target volume (PTV) was defined as the CTV plus an additional margin of 0.5 cm. According to the tumor location and physician discretion, all patients were irradiated in a total dose of more than 50.0 Gy with 1.8–2.2 Gy per fraction and 5 fractions per week. Patients received concurrent chemotherapy (cisplatin or nedaplatin-based regimen) every 3 weeks during radiotherapy for up to five cycles. 4 patients received weekly docetaxel and cisplatin for four or five cycles to alleviate toxic side effects considering the patient's physical condition.

### Follow-up

After treatment, all patients received weekly examinations for toxicities during IC or CCRT, such as complete blood count, biochemistry, etc. Patients were re-evaluated for acute side effects such as barium esophagography and complete blood count 1 month after treatment completion, then physical examination, CT scanning of the neck, chest and abdomen, and ultrasound were performed every 3 months during the first 2 years, every 6 months from the second to the fifth year, and annually thereafter. Information about survival status and disease progression was updated until April 2023. The endpoints of the study were OS, recurrence-free survival (RFS), and distant metastasis-free survival (DMFS). OS was calculated from the date of diagnosis to death or last follow-up. RFS was calculated from the date of treatment to locoregional recurrence or death. DMFS was calculated from the date of treatment to distant metastasis or death. Toxicity was assessed according to the National Cancer Institute Common Terminology Criteria for Adverse Events version 4.0 (CTCAE v4.0).

### Statistical analysis

The clinical tumor response was assessed 2 weeks after IC by enhanced CT scans and barium swallow according to the Response Evaluation Criteria in Solid Tumor criteria 1.1 (RECIST)^18^, which is divided into four grades (complete response [CR], partial response [PR], stable disease [SD], and progressive disease [PD]). Patients were categorized into the following two groups: patients who achieved CR, PR, and SD (IC responders group) and patients who showed PD (IC non-responders group after IC. We also defined CR/PR as “IC good responders” and SD/PD as “IC poor responders”.

All statistical tests for data analysis were performed using Statistical Analysis System (SAS) version 9.4. The PSM was performed to reduce the effect of treatment selection bias. A 1:1 matching of CCRT to IC-CCRT patients was generated based on several factors such as age, gender, primary tumor location, T stage, N stage, and initial clinical stage using the nearest neighbour method at a calliper of 0.6. Survival curves were estimated by use of the Kaplan-Meier method and groups were compared for their survival rates by the log-rank test. Both univariate and multivariate analysis were performed by use of Cox regression models to identify significant prognostic factors. Hazard ratios (HRs) and 95% confidence intervals (CIs) were estimated for each prognostic factor. A p-value of < 0.05 was considered to be statistically significant.

## Results

### Baseline characteristics

In total, the clinical data of 271 newly diagnosed ESCC patients were collected and retrospectively reviewed. From the original data, 71 pairs were selected by the PSM method (Supplementary Figure 1). The baseline characteristics of the patients are summarized in Supplementary Table 1. For the selected subject, the median age was 61.5 years (range 38–74 years), and the study population included 115 (81.0%) males and 27 (19.0%) females. Among the patients, 118 patients (83.1%) had T3 or T4 disease, and 126 (88.7%) had lymph node metastasis. 30 patients (21.2%) had stage I or II disease and 112 (78.9%) had stage III or IV. The median tumor length was 6 cm (range, 2–23 cm). The median total radiation dose was 59.36 Gy (range, 50.4–66.0 Gy). The median follow-up time of the study was 21 months. There were no statistically significant differences in age, gender, zubrod performance status (ZPS) score, tumor length, and stages between the CCRT group and IC + CCRT group.

### Survival outcomes

**Figure 1.**
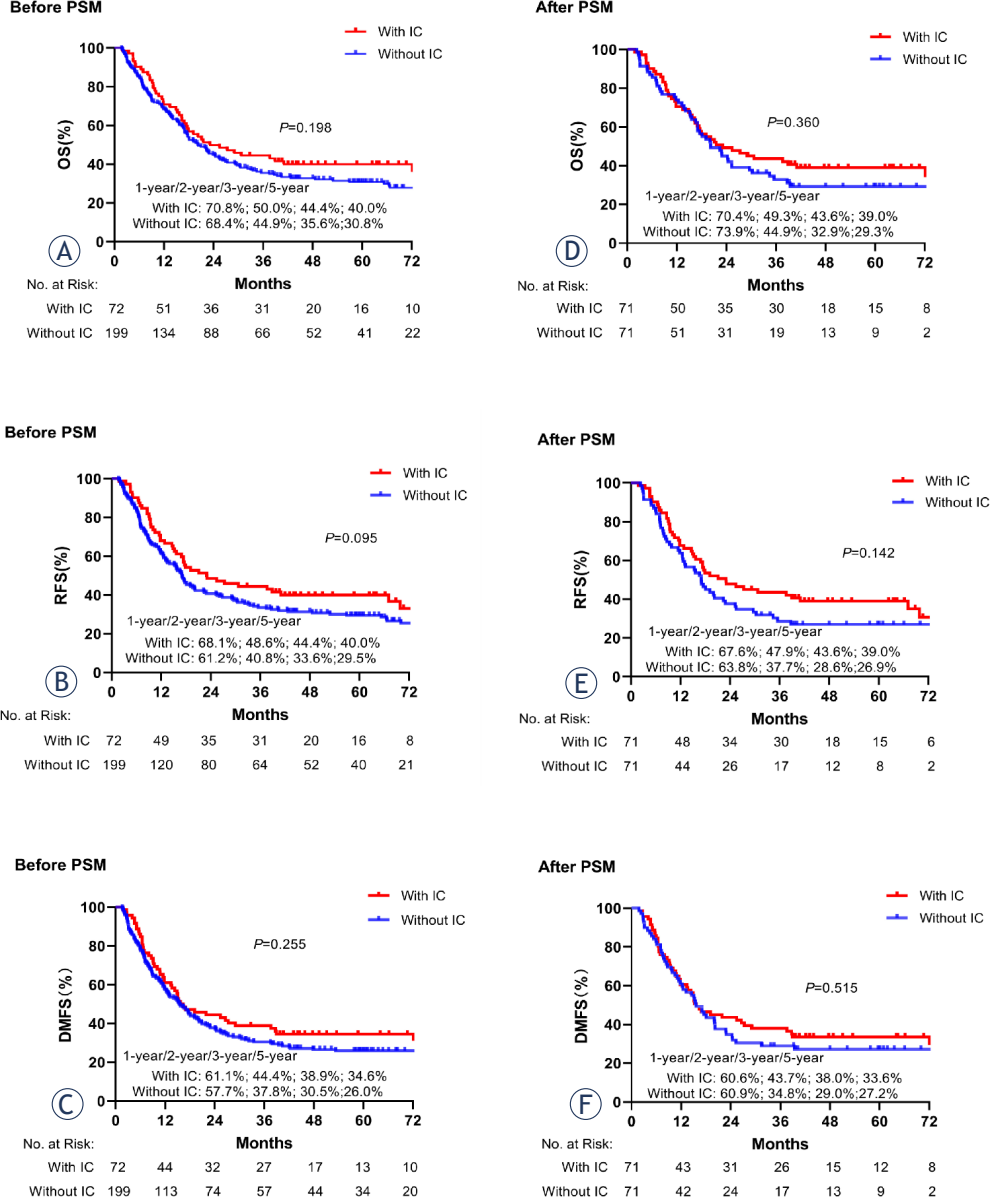
Kaplan-Meier survival curves of the induction chemotherapy (IC) + concurrent chemoradiotherapy (CCRT) group and CCRT group in patients before and after matching. **(A, D)** overall survival (OS); **(B, E)** recurrence-free survival (RFS); **(C, F)** distant metastasis-free survival (DMFS). PSM = propensity score matching

In the original data set (n = 271), survival outcomes were similar and non-significant between the CCRT group and IC + CCRT group (p > 0.05, Figure 1A–C). In terms of the matched data set, the IC + CCRT group achieved a tendency for improvement in 3-, and 5-year OS rate (43.6% *vs.* 32.9%, 39.0% *vs.* 29.3%, p = 0.360; Figure 1D), RFS (43.6% *vs.* 28.6%, 39.0% *vs.* 26.9%, p = 0.142; Figure 1E), and DMFS (38.0% *vs.* 29.0%, 33.6% *vs.* 27.2%, p = 0.515; Figure 1F) compared with the CCRT group, although the difference between the two groups did not reach statistical significance. Details regarding the reasons for death are provided in Supplementary Table1. The most common reasons for death were dysphagia, metastasis or recurrence, and gastrointestinal bleeding (IC + CCRT *vs.* CCRT: 15.5% *vs.* 21.1%, 28.2% *vs.* 29.6%, 11.3% *vs.* 5.6%, respectively), and no statistically significant differences were not found between the two groups.

### Prognostic factors

**Figure 2.**
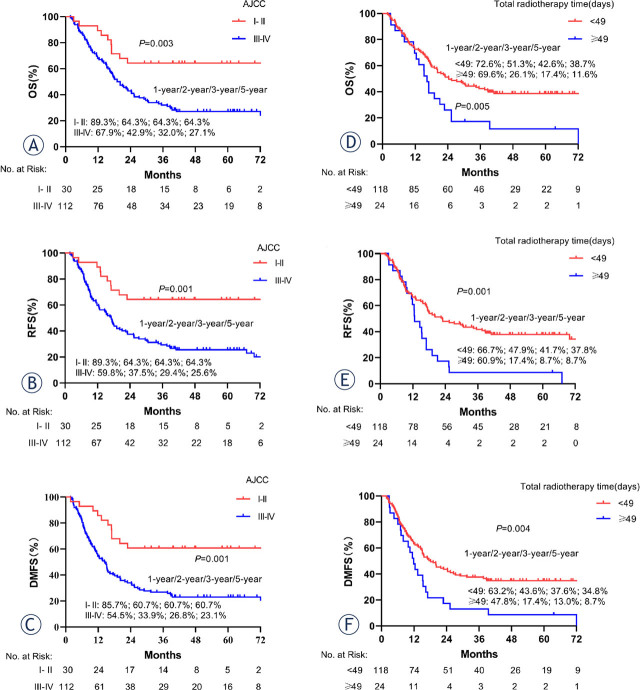
Kaplan-Meier survival curves based on the American Joint Committee on Cancer (AJCC) stage and total radiotherapy time for the propensity-matched cohort. **(A, D)** overall survival (OS); **(B, E)** recurrence-free survival (RFS); **(C, F)** distant metastasis-free survival (DMFS).

We included both demographic and clinicopathologic variables in the univariate analysis (Table 2 and Supplementary Table 2). Total radiotherapy time, age, and the 8th edition of the American Joint Committee on Cancer (AJCC) stage were identified as significant predictive factors of prognosis by multivariate analysis (Table 3 and Supplementary Table 3). Briefly, the total radiotherapy time ≥ 49 days and AJCC stage III/IV were independently associated with worse OS (p = 0.025, HR = 1.762, 95% CI = 1.074–2.891; p = 0.006, HR = 2.533, 95% CI = 1.305–4.916), RFS (p = 0.009, HR = 1.920, 95% CI = 1.178–3.131; p = 0.003, HR = 2.738, 95% CI = 1.413–5.305) and DMFS (p = 0.014, HR = 1.827, 95% CI = 1.127–2.961; p = 0.001, HR = 2.951, 95% CI = 1.560–5.582). Besides, age ≥ 60 (p = 0.011; HR, 0.592; 95% CI, 0.396–0.886) was independently associated with better DMFS (Supplementary Figure 2). As shown in Figure 2, there was a significant difference in OS, RFS, and DMFS in total radiotherapy time and AJCC stage.

**Table 1.** Baseline characteristics for patients before and after propensity score matching (PSM) [M (QL, QU)/n(%)]

**Variables**	**Before PSM (n = 271)**				**After PSM (n = 142)**			

	**Total**	**Without IC (n = 199)**	**With IC (n = 72)**	**P**	**Total**	**Without IC (n = 71)**	**With IC (n = 71)**	**P**
Age (year)	61.0 (56.0, 65.0)	61.0 (56.0, 66.0)	61.5 (55.0, 65.0)	0.739	61.5 (56.0, 65.0)	61.0 (57.0, 65.0)	62.0 (55.0, 65.0)	0.923
Total radiotherapy time (day)	43.0 (40.0, 47.0)	43.0 (40.0, 48.0)	42.0 (40.0, 45.8)	0.226	43.0 (40.0, 46.3)	43.0 (39.0, 48.0)	42.0 (40.0, 46.0)	0.361
Age (year)				0.820				0.865
< 60	116 (42.8)	86 (43.2)	30 (41.7)		59 (41.5)	30 (42.3)	29 (40.8)	
≥ 60	155 (57.2)	113 (56.8)	42 (58.3)		83 (58.5)	41 (57.7)	42 (59.2)	
Gender				0.073				0.285
Female	62 (22.9)	51 (25.6)	11 (15.3)		27 (19.0)	16 (22.5)	11 (15.5)	
Male	209 (77.1)	148 (74.4)	61 (84.7)		115 (81.0)	55 (77.5)	60 (84.5)	
ECOG PS				<0.001				1.000
0–1	183 (67.5)	148 (74.4)	35 (48.6)		70 (49.3)	35 (49.3)	35 (49.3)	
2–3	88 (32.5)	51 (25.6)	37 (51.4)		72 (50.7)	36 (50.7)	36 (50.7)	
Tumor Length(cm)				0.540				0.851
< 8	203 (74.9)	151 (75.9)	52 (72.2)		103 (72.5)	52 (73.2)	51 (71.8)	
≥ 8	68 (25.1)	48 (24.1)	20 (27.8)		39 (27.5)	19 (26.8)	20 (28.2)	
T stage				0.739				0.179
1–2	37 (13.7)	28 (14.1)	9 (12.5)		24 (16.9)	15 (21.1)	9 (12.7)	
3–4	234 (86.3)	171 (85.9)	63 (87.5)		118 (83.1)	56 (78.9)	62 (87.3)	
N stage				0.181				1.000
0–1	204 (75.3)	154 (77.4)	50 (69.4)		100 (70.4)	50 (70.4)	50 (70.4)	
2–3	67 (24.7)	45 (22.6)	22 (30.6)		42 (29.6)	21 (29.6)	21 (29.6)	
AJCC stage				0.291				0.411
I–II	61 (22.5)	48 (24.1)	13 (18.1)		30 (21.1)	17 (23.9)	13 (18.3)	
III–IV	210 (77.5)	151 (75.9)	59 (81.9)		112 (78.9)	54 (76.1)	58 (81.7)	
IC cycles (times)				<0.001				< 0.001
0	199 (73.4)	199 (100.0)	0 (0)		71 (50.0)	71 (100.0)	0 (0)	
1/2	56 (20.7)	0 (0)	56 (77.8)		56 (39.4)	0 (0)	56 (78.9)	
3/4	16 (5.9)	0 (0)	16 (22.2)		15 (10.6)	0 (0)	15 (21.1)	
Response after IC								
CR	—	—	0 (0)		—	—	0 (0)	
PR	—	—	32 (44.4)		—	—	32 (45.1)	
SD	—	—	33 (45.8)		—	—	32 (45.1)	
PD	—	—	7 (9.7)		—	—	7 (9.9)	

AJCC stage = American Joint Committee on Cancer stage; Adjusted factors = age, gender, ECOG PS, tumor length, T stage, N stage; CR = complete response; ECOG PS = Eastern Cooperative Oncology Group performance status; IC = induction chemotherapy; PD = progressive disease; PR = partial response; PD = stable disease

**Table 2.** Univariate Cox analysis of overall survival (OS), recurrence-free survival (RFS), and distant metastasis-free survival (DMFS) after propensity score matching (PSM)

**Variables**	**OS**		**RFS**		**DMFS**	

	**HR (95% CI)**	**P**	**HR (95% CI)**	**P**	**HR (95% CI)**	**P**
Age (year)	0.986 (0.958–1.014)	0.324	0.985 (0.957–1.014)	0.306	0.981 (0.954–1.008)	0.161
Total radiotherapy time (day)	1.018(1.001–1.034)	0.032	1.015(1.000–1.031)	0.051	1.016(1.001–1.031)	0.039
Age (year)						
< 60	1.000		1.000		1.000	
≥ 60	0.749 (0.498–1.128)	0.167	0.794 (0.530–1.191)	0.265	0.682 (0.459–1.015)	0.059
Gender						
Female	1.000		1.000		1.000	
Male	1.566 (0.886–2.766)	0.122	1.511 (0.870–2.624)	0.143	1.808 (1.026–3.186)	0.040
ECOG PS						
0–1	1.000		1.000		1.000	
2–3	1.058 (0.701–1.596)	0.788	1.121 (0.747–1.682)	0.580	1.125 (0.755–1.675)	0.564
Tumor Length(cm)						
< 8	1.000		1.000		1.000	
≥ 8	1.481 (0.954–2.301)	0.080	1.651 (1.068–2.553)	0.024	1.510 (0.985–2.314)	0.059
Total radiotherapy time (day)						
< 49	1.000		1.000		1.000	
≥ 49	2.018 (1.234–3.300)	0.005	2.203 (1.354–3.583)	0.001	2.016 (1.249–3.255)	0.004
T stage						
1–2	1.000		1.000		1.000	
3–4	2.938 (1.421–6.075)	0.004	3.162 (1.530–6.536)	0.002	2.984 (1.501–5.932)	0.002
N stage						
0–1	1.000		1.000		1.000	
2–3	1.150 (0.744–1.779)	0.529	1.227 (0.798–1.886)	0.352	1.262 (0.827–1.926)	0.280
AJCC stage						
I–II	1.000		1.000		1.000	
III–IV	2.751 (1.426–5.307)	0.003	2.983 (1.548–5.752)	0.001	2.940 (1.568–5.511)	0.001
IC cycles (times)						
0	1.000		1.000		1.000	
1/2	0.935 (0.609–1.435)	0.759	0.835 (0.546–1.275)	0.403	0.921 (0.606–1.399)	0.699
3/4	0.509 (0.230–1.127)	0.096	0.458 (0.207–1.012)	0.054	0.725 (0.356–1.475)	0.374

Hazard ratios and 95% confidence intervals were calculated by a stratified Cox proportional hazards model.

AJCC stage = American Joint Committee on Cancer stage; ECOG PS = Eastern Cooperative Oncology Group performance status; IC = induction chemotherapy

**Table 3.** Cox multivariate analysis of overall survival (OS), recurrence-free survival (RFS), and distant metastasis-free survival (DMFS) after propensity score matching (PSM)

**Variables**	**OS**		**RFS**		**DMFS**	

	**HR (95% CI)**	**P**	**HR (95% CI)**	**P**	**HR (95% CI)**	**P**
Total radiotherapy time (day)						
< 49	1.000		1.000		1.000	
≥ 49	1.762 (1.074–2.891)	0.025	1.920 (1.178–3.131)	0.009	1.827 (1.127–2.961)	0.014
AJCC stage						
I–II	1.000		1.000		1.000	
III–IV	2.533 (1.305–4.916)	0.006	2.738 (1.413–5.305)	0.003	2.951 (1.560–5.582)	0.001
Age (year)						
< 60					1.000	
≥ 60					0.592 (0.396–0.886)	0.011

Hazard ratios and 95% confidence intervals were calculated by a stratified Cox proportional hazards model.

AJCC stage = American Joint Committee on Cancer stage

### Subgroup analysis

**Figure 3.**
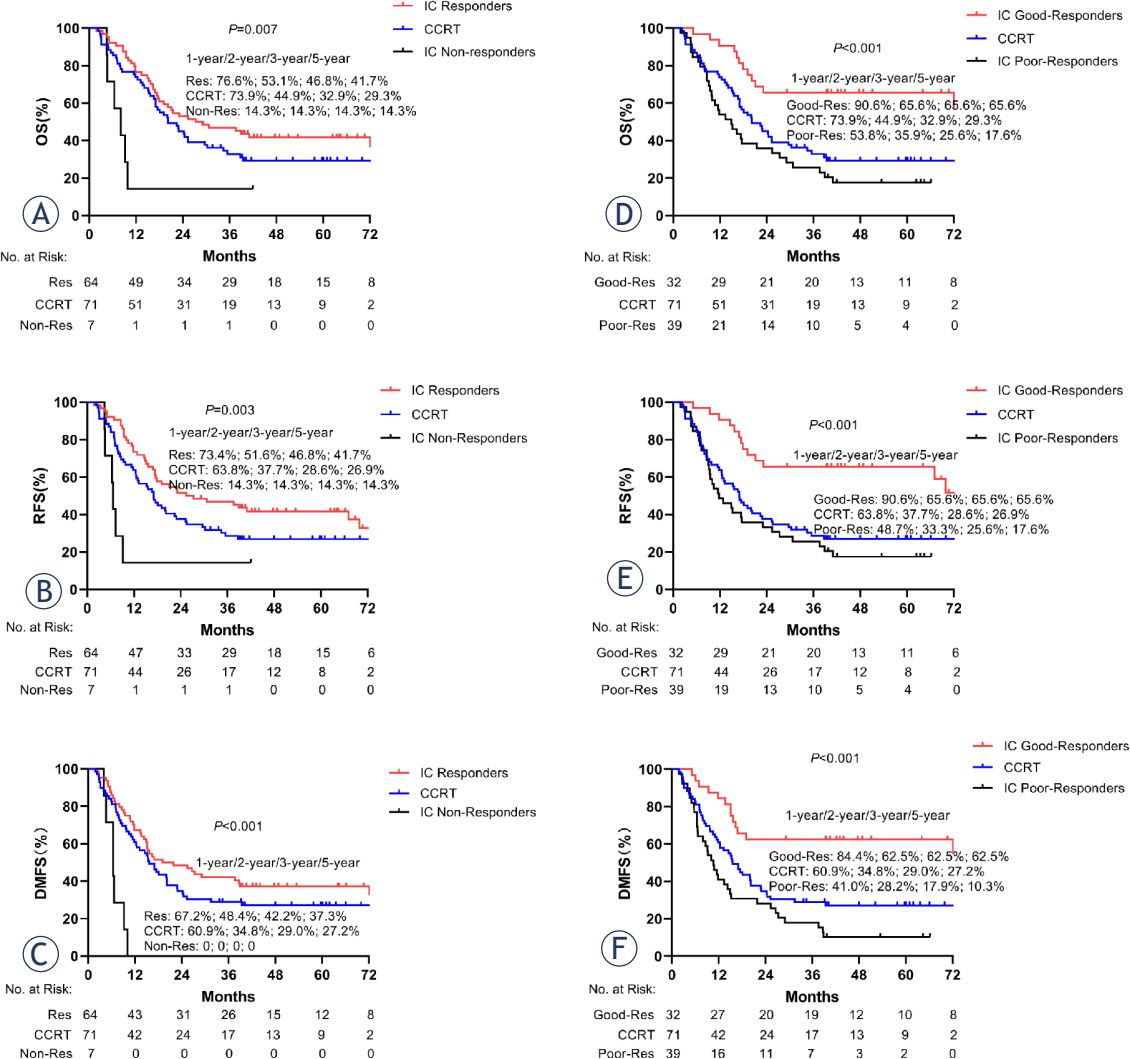
Kaplan-Meier estimates of survival curves based on the clinical response to induction chemotherapy. **(A–C)** Induction chemotherapy (IC) non-responders group *vs.* the IC responders group *vs.* the concurrent chemoradiotherapy (CCRT) group. **(D–F)** IC good-responders group *vs.* the IC poor-responders group versus the CCRT group.

Tumor responses after IC are listed in Table 1. After IC, CR was obtained in 0 patients (0%), PR in 32 (45.1%), SD in 32 (45.1%), and PD in 7 patients (9.9%), respectively. For 71 patients with IC, the overall response rate (CR + PR + SD), and good response rate (CR + PR) were 90.1%, and 45.1%, respectively. The potential effect of tumor response to IC on survival outcomes was also analysed as a predictive factor. As shown in Figure 3, the responders to IC had significantly more favourable survival compared with non-responders, or with patients in the CCRT group, with corresponding 5-year OS rates of 41.7%, 14.36%, and 29.3%, 5-year RFS rates of 41.7%, 14.3%, and 26.9%, and 5-year DMFS rates of 37.3%, 0%, and 27.2%, respectively (p < 0.001 for OS, RFS and DMFS, Figure 3A–C). Likewise, the 5-year OS rates (65.6% *vs.* 17.6% *vs.* 29.3%, p < 0.001; Figure 3D), RFS rates (65.6% *vs.* 17.6% *vs.* 26.9%, p < 0.001; Figure 3E), and DMFS rates (62.5% *vs.* 10.3% *vs.* 27.2%, p < 0.001; Figure 3F) of the IC good responders were significantly higher than that of the IC poor responders and CCRT group. We also studied the survival outcomes and response to IC for different IC regimens. The results showed that there was no significant statistical difference between different IC regimens and OS, RFS, and DMFS (p > 0.05) (Supplementary Figure 3). No significant differences were found between different IC regimens and good responders (Supplementary Table 4).

**Figure 4.**
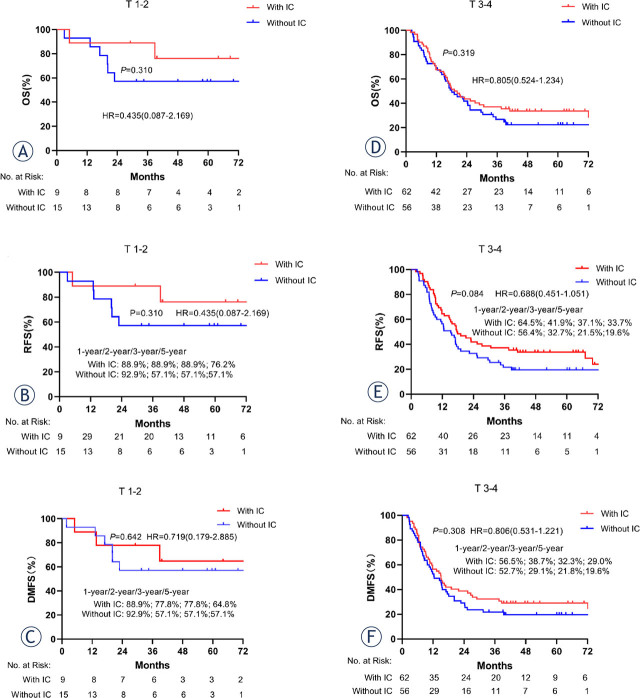
Kaplan-Meier estimates of survival curves based on the T stage. **(A–C)** overall survival (OS), recurrence-free survival (RFS), and distant metastasis-free survival (DMFS) of patients with T1–2; **(D–F)** OS, RFS, and DMFS of patients with T3–4. IC = induction chemotherapy

To further distinguish the survival difference in patients on different risk stratification, a subgroup analysis was performed according to the T stage. In the subgroup of patients with T3–4 ESCC disease, 62 and 55 cases receiving IC + CCRT and CCRT, respectively, were selected for subgroup analysis. Compared with the CCRT group, the IC + CCRT group achieved better 5-year OS (33.7% *vs.* 22.5%, p = 0.319; Figure 4D), RFS (33.7% *vs.* 19.6%, p = 0.084; Figure 4E), and DMFS (29.0% *vs.* 19.6%, p = 0.308; Figure 4F), however, there was no significant difference. In addition, IC + CCRT showed a similar tendency for 5-year OS (76.2% *vs.* 57.1%, p = 0.310; Figure 4A), RFS (76.2% *vs.* 57.1%, p = 0.310; Figure 4B), and DMFS (64.8% *vs.* 57.1%, p = 0.642; Figure 4C) versus CCRT in the T1–2 subgroup.

### Failure mode

Patterns of treatment failure in ESCC patients are listed in Table 4. In terms of the matched data set, locoregional failure occurred in 12 patients (16.9%) and 11 patients (15.5%) in the IC + CCRT group and CCRT group, respectively. In the IC + CCRT group, 22 patients (31%) experienced distant failure, and in the CCRT group 19 patients experienced distant failure.

**Table 4.** Failure pattern [n (%)]

**Variables**	**Before PSM**			**After PSM**		

	**Without IC (n=199)**	**With IC (n=72)**	**p**	**Without IC (n=71)**	**With IC (n=71)**	**p**
Local and/or regional						
Local only	26 (13.1)	10 (13.9)	0.860	12 (16.9)	10 (14.1)	0.643
Local and regional	2 (1.0)	1 (1.4)	1.000	0 (0)	1 (1.4)	1.000
Regional only	0 (0)	0 (0)	—	0 (0)	0 (0)	—
Total locoregional failure	28 (14.1)	11 (15.3)	0.802	12 (16.9)	11 (15.5)	0.820
Distant						
Bone only	3 (1.5)	1 (1.4)	1.000	1 (1.4)	1 (1.4)	1.000
Liver only	7 (3.5)	3 (4.2)	1.000	4 (5.6)	3 (4.2)	1.000
Lung only	17 (8.5)	5 (6.9)	0.670	6 (8.5)	5 (7.0)	0.754
Brain only	1 (0.5)	1 (1.4)	1.000	0 (0)	1 (1.4)	1.000
Multiple location^a^	10 (5.0)	6 (8.3)	0.466	4 (5.6)	7 (9.9)	0.346
Other location^b^	19 (9.5)	6 (8.3)	0.760	4 (5.6)	5 (7.0)	1.000
Total distant failure	57 (28.6)	22 (30.6)	0.760	19 (26.8)	22 (31.0)	0.579

a Combinations of bone, brain, liver, lung, and lymph nodes;

b including pleural and lymph nodes

IC = induction chemotherapy; PSM = propensity score matching

### Toxicities

During induction chemotherapy, leucopenia was the most common adverse event (Supplementary Table 5), which was observed in 32 patients (45.1%). Patients developed grade 3 or 4 hematologic toxicity including neutropenia (n = 9, 12.7%), leucopenia (n = 9, 12.7%), febrile neutropenia (n = 1, 1.4%), and anemia (n = 1, 1.4%).

Over the entire treatment phase, grade 3–4 radiation esophagitis (RE) was identified in 2.8% (2/71) of the IC + CCRT group and 4.2% (3/71) of the CCRT group (p = 0.678). Hematologic toxicity grade 3-4 was observed in 19 (26.8%) and 20 (28.2%) patients who received IC + CCRT and CCRT alone, respectively (p = 0.944). Although 43.7% of patients (31/71) developed esophageal stricture in the IC + CCRT group, the incidence of grade 3-4 adverse events was only 4.2% (3/71), which was not serious. No significant differences were observed in the rates of other grades 3-4 toxicities between both groups (Table 5).

**Table 5.** Acute and late toxicities during treatment before and after propensity score matching (PSM) [n (%)]

**Toxicities**	**Before PSM**				**p**	**After PSM**				**p**
	
	**Without IC (n=199)**		**With IC (n=72)**			**Without IC (n=71)**		**With IC (n=71)**		
	
	**Grade 1-2**	**Grade 3-4**	**Grade 1-2**	**Grade 3-4**		**Grade 1-2**	**Grade 3-4**	**Grade 1-2**	**Grade 3-4**	
Acute adverse events										
Esophagitis	183 (92.0)	6 (3.0)	67 (93.1)	2 (2.8)	0.951	63 (88.7)	3 (4.2)	66 (93.0)	2 (2.8)	0.678
Myelosuppression	109 (54.8)	58 (29.1)	40 (55.6)	19 (26.4)	0.873	37 (52.1)	20 (28.2)	39 (54.9)	19 (26.8)	0.944
Radiation pneumonitis	1 (0.5)	0 (0)	1 (1.4)	0 (0)	1.000	0 (0)	0 (0)	1 (1.4)	0 (0)	1.000
Esophageal fistula	3 (1.5)	0 (0)	0 (0)	0 (0)	0.696	0 (0)	0 (0)	0 (0)	0 (0)	—
Late adverse events										
Esophageal stricture	92 (46.2)	3 (1.5)	29 (40.3)	3 (4.2)	0.364	29 (40.8)	1 (1.4)	28 (39.4)	3 (4.2)	0.584

Acute adverse events: ≤ 3 months after completion of study treatment; Late adverse events: > 3 months after study treatment

IC = induction chemotherapy

## Discussion

The efficacy of IC has not been well documented previously for ESCC patients receiving IMRT/VMAT-based CCRT. In the present study, we performed a PSM analysis of patients treated with or without IC before standard CCRT to better understand the efficacy and toxicities of IC. We found that IC + CCRT was not superior to CCRT in terms of 5-year OS, RFS, and DMFS regarding original or well-matched data. The stratified analysis further demonstrated IC + CCRT improved the 5-year OS, RFS, and DMFS for the patients with response (responders or good responders) to IC, whereas it might not have a positive impact for non-responding or poorly responding patients and seemed to have limited benefits in long-term survival. Nearly all of the patients who are alive in our study have completed valid follow-up for 2 years (except for individual deleted data), and the longest followup period was over 7 years. Concerning toxicity, there was no significant difference in toxicity between patients who had IC and those who did not. According to our knowledge, this is the first study to compare the survival benefits of the addition of IC to CCRT and IMRT/VMAT only in ESCC patients. The main strength of our study is that the application of the PSM method balances the baseline characteristics of the included population to reduce potential confounders, thus mimicking the matching observed in randomized controlled trials (RCTs).

Since the Radiation Therapy Oncology Group (RTOG) 85–01 trial indicated that the outcome of CCRT was significantly better than that of RT alone for ESCC patients, definitive CCRT has been a standard treatment.^5^ However, the long-term outcomes remain limited and the failure rate was 50%.^6,7^ Updated meta-analyses and systematic reviews of clinical trials have demonstrated that IC + CCRT could prolong short-term survival in unresectable EC patients.^19^ Unfortunately, several subsequent similar studies including a prospective randomized clinical trial got negative results.^9,11,12,13^ In our study, we found that the IC + CCRT group achieved higher 5-year OS (39.0% *vs.* 29.3%, p = 0.360), RFS (39.0% *vs.* 26.9%, p = 0.142), and DMFS (33.6% *vs.* 27.2%, p = 0.515) compared with the CCRT group, although the difference between the two groups did not reach statistical significance. Rational explanations for the discrepancy may include: 1) The enrolled patients in the current study included clinical stages T1–4N0–3, and the patients with early-stage disease (T1–2N0–1) may not benefit from IC. 2) The regimens of IC in the retrospective studies were not uniform, which may provide a slight bias toward a negative result.

It was reported that the late tumor stage was an important risk factor for poor prognosis in ESCC.^20^ In this study, multivariate Cox analysis showed AJCC stage was regarded as an independent predictive factor that affects OS, RFS, and DMFS. Later AJCC stage has inferior OS (p = 0.006; HR, 2.533; 95% CI, 1.305–4.916) and RFS (p = 0.003; HR, 2.738; 95% CI, 1.413–5.305), and DMFS (p = 0.001; HR, 2.951; 95% CI, 1.560–5.582) than early AJCC stage ESCC, which was similar to those in Hsieh's report.^21^ We also found radiotherapy treatment time was another independent predictive factor for OS, RFS, and DMFS. In daily clinical practice, unplanned treatment interruptions are inevitable for many reasons. Sher reported that a prolonged total radiotherapy time > 51 days is associated with an inferior overall survival (hazard ratio = 1.63, p = 0.0058). For each additional day required to finish radiotherapy, the hazard rate of death increased by 4.2%.^22^ Cannon *et al.* reviewed outcomes of 171 head and neck cancer patients treated with CCRT and found that patients with radiotherapy time ≤ 49 days had a superior 3-year local control rate and OS compared to those with radiotherapy time > 49 days (88% versus 71%, 81% versus 58%, respectively).^23^ In our study, the total radiotherapy time ≥ 49 days has the inferior OS (p = 0.025; HR, 1.762; 95% CI, 1.074–2.891), RFS (p = 0.009; HR, 1.920; 95% CI, 1.178–3.131) and DMFS (p = 0.014; HR, 1.827; 95% CI, 1.127–2.961). For patients with total radiotherapy time ≥ 49 days in our study, the vast majority of interruption was due to machine breakdown, machine maintenance, and treatment toxicity (chemoradiotherapy or radiotherapy only). Briefly, 11 patients experienced treatment breaks because of radiation esophagitis. Meanwhile, 6 patients experienced unscheduled interruptions due to chemotherapy toxicities. Therefore, it seems a necessity for radiotherapy without gaps or delays for the sake of improved outcomes and control of disease progression.

So far, there is no evidence to suggest that advanced age is an independent contraindication for CCRT in the retrospective studies.^20, 24^ Wu *et al.* reported that there was no statistically significant difference between CCRT and RT alone for patients aged 75 years or older.^20^ In the present study, we found that age is not a predictive factor of OS (p = 0.167; HR, 0.749; 95% CI, 0.498–1.128) and RFS (p = 0.265; HR, 0.794; 95% CI, 0.530–1.191) by multivariate analysis. Interestingly, we found that age < 60 was independently associated with worse DMFS in the current study.^25^ Similar results have been found in Colzani'study, suggesting that breast cancer patients younger than 50 years at diagnosis had a higher risk of distant metastasis.^26^ The possible reason may be that the metastases lose aggressive character or that the host defense is better equipped to deal with them in advancing age.^27^

IC may only benefit a certain subgroup but not unselected patients with ESCC. As is known to all, the T stage was associated with a worse prognosis in esophageal carcinoma.^28^ Akinori reported that IC for T4 esophageal cancer offered comparable local control and survival to conventional CCRT, and suggested that the strategy of IC followed by CCRT was efficient for T4M0 esophageal cancer.^13^ To identify the subgroups that may benefit from IC, we performed a stratified analysis based on the T stage. Our results indicated that IC + CCRT group achieved better 5-year OS (33.7% *vs.* 22.5%), RFS (33.7% *vs.* 19.6%), and DMFS (29.0% *vs.* 19.6%) compared with the CCRT group, however, there was no significant difference. Moreover, 90% of the symptoms of dysphagia improved significantly after IC, which was consistent with the trial INT 0122.^29^ In the study by Luo *et al.*^12^, the IC responders (CR or PR) group achieved significantly more favourable OS compared with the IC non-responders (SD or PD) group and the CCRT alone group (p = 0.002). Besides, the post-hoc analysis in prospective research also demonstrated that response to IC was associated with more favourable survival.^9^ Consistent with the results of previous studies, our results suggested that the responders (CR, PR, or SD) to IC had significantly more favourable survival compared with non-responders (PD), or with patients in the CCRT group, with corresponding 5-year OS rates of 41.7%, 14.3%, and 29.3%, respectively. In addition, we further analyzed the potentially important role of IC good responders (CR or PR) from the whole IC group. Our data suggested that the IC good responders might have a significantly prolonged OS, improved locoregional control, and reduced distant metastasis, with corresponding 5-year OS rate, RFS rate, and DMFS of 65.6%, 65.6%, and 62.5%, respectively. Considering the poor prognosis of the IC non-responders or IC poor responders, tumor response after IC could be used to guide subsequent treatment decisions, such as switching to alternative agents included targeted therapies, immunotherapies, or radiosensitizers during radiotherapy for non-responders or IC poor responders. Therefore, further studies are needed to overcome this unfavorable biological characteristic.

In our study, the rate of grade ≥ 3 RE in the IC + CCRT group and the CCRT group had no statistical significance (p = 0.678). There was also no statistical difference in the incidence of myelosuppression (p = 0.944). It has been reported that patients with T4 had an incidence of perforation of 14–23% during CCRT, and the addition of IC before CCRT might reduce the risk of perforation by decreasing the tumor volume before encountering severe esophagitis.^30,31^ No esophageal fistula or perforation occurred in our study, which is one of the most troublesome complications caused by CCRT. The possible reason was that ESCC patients by using IMRT/VMAT only are superior to the two-dimensional conformal radiation (2D-CRT) or 3D-CRT. IMRT/VMAT improves the treatment ratio due to the highly conformal dose distributions in the tumor target volume and sharp dose gradients at the transition to the adjacent normal structures. The potential benefits of IMRT/VMAT were investigated in a series of studies.^32^ Our results are consistent with the outcomes of the studies in the IMRT/VMAT era.

There were several limitations in our study. First, although we used PSM, a method aimed to minimize the impact of observed confounders, the retrospective nature of this study cannot exclude the possibility of bias caused by confounding factors, and adding too many match restrictions would lead to small sample size and might not represent the initial population. Secondly, our study is limited to ESCC patients and could not applied to other types of EC. Finally, due to the retrospective characteristic, IC regimes and concurrence chemotherapy regimens were not uniform. More well-designed prospective, randomized controlled trials are warranted to further confirm the role of IC.

## Conclusions

In this study, our results showed the addition of IC to CCRT was not superior to CCRT in unselected ESCC patients, while IC responders might benefit from this regime without an increase in toxicities.

## Supplementary Material

Supplementary Material Details
