# Supplementary material for: Long-term results of induction chemotherapy for non-operable esophageal squamous cell carcinoma followed by concurrent chemoradiotherapy: a single-centre experience
Source: Radiol Oncol. 2024 Sep 15;58(3):444–57. doi: 10.2478/raon-2024-0038 (PMC11406992; doi:10.2478/raon-2024-0038)
Supplement: Supplementary file 1 — Supplementary Material Details [file raon-2024-0038-sm.pdf]

Long-term outcome of induction chemotherapy followed by concurrent chemoradiotherapy for non-operable esophageal squamous cell carcinoma: a single-center experience

Geng Xiang, Guangjin Chai, Bo Lyu, Zhaohui Li, Yutian Yin, Bin Wang, Yanglin Pan, Mei Shi, Lina Zhao

doi: 10.2478/raon-2024-0038

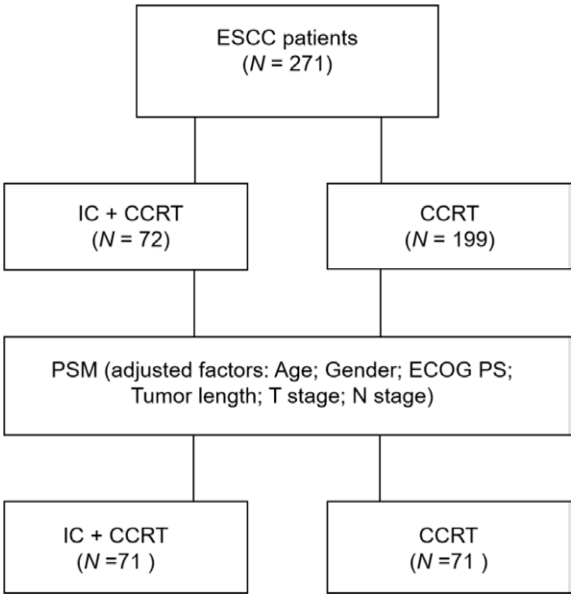

SUPPLEMENTARY FIGURE 1. Flow chart of patient selection.

CCRT = concurrent chemoradiotherapy; ECOG PS = Eastern Cooperative Oncology Group performance status; ESCC = esophageal squamous cell carcinoma; IC = induction chemotherapy; PSM = propensity score matching

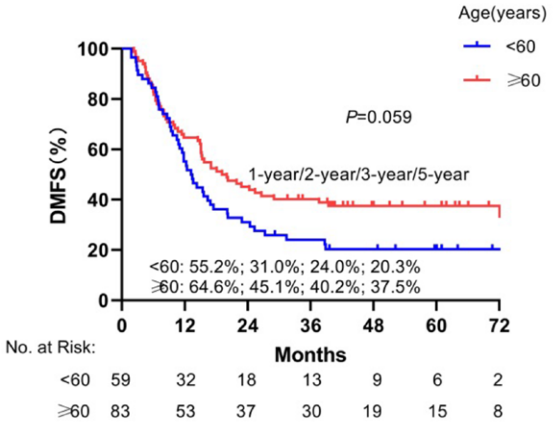

SUPPLEMENTARY FIGURE 2. Kaplan-Meier analysis of distant metastasis-free survival (DMFS) according to age after matching.

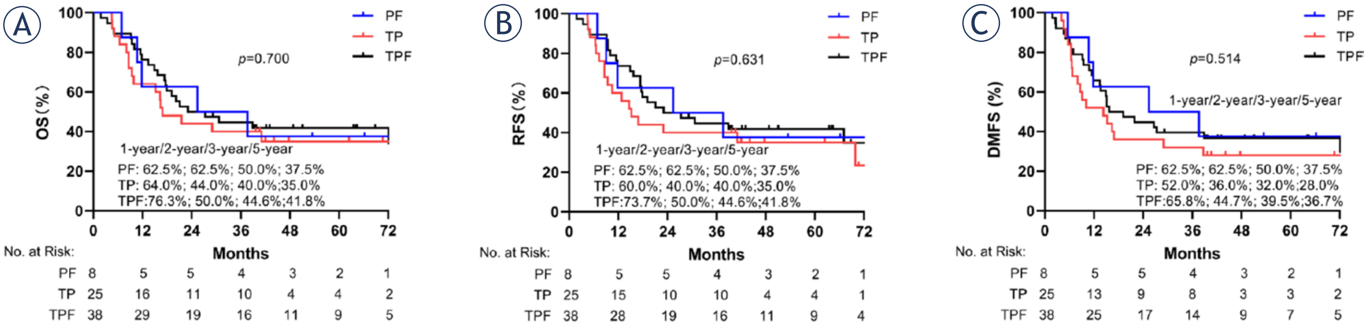

SUPPLEMENTARY FIGURE 3. Kaplan-Meier survival curves based on different chemotherapy regimens. **(A)** overall survival (OS); **(B)** recurrence-free survival (RFS); **(C)** distant metastasis-free survival (DMFS).

PF = cisplatin 50 mg/m2/day on days 1 to 2 and 5-fluorouracil 500 mg/m2/day on days 1 to 3;

TP = docetaxel 60 mg/m2/day on day 1 or paclitaxel 150 mg/m2/day on day 1 and day 8, cisplatin 50 mg/m2/day on days 1 to 2);

TPF = docetaxel 60 mg/m2/day on day 1, cisplatin 50 mg/m2/day on days 1 to 2, and 5-fluorouracil 500 mg/m2/day on days 1 to 3

SUPPLEMENTARY TABLE 1. Cause of death before and after propensity score matching (PSM) [n(%)]

Cause	Before PSM			After PSM		
	Without IC (n = 199)	With IC (n = 72)	*P*	Without IC (n = 71)	With IC (n = 71)	*P*
Dysphagia	33(16.6)	11(15.3)	0.194	15(21.1)	11(15.5)	0.307
Metastasis or recurrence	57(28.6)	20(27.8)		21(29.6)	20(28.2)	
Gastrointestinal bleeding	20(10.1)	8(11.1)		4(5.6)	8(11.3)	
Esophageal fistula or perforation	2(1.0)	3(4.2)		1(1.4)	3(4.2)	
Radiation pneumonitis	8(4.0)	0(0)		4(5.6)	0(0)	
Others	19(9.5)	3(4.2)		3(4.2)	3(4.2)	

IC = induction chemotherapy;

SUPPLEMENTARY TABLE 2. Univariate Cox analysis of overall survival (OS), recurrence-free survival (RFS), before propensity score matching (PSM)

Variables	OS		RFS		DMFS	
	HR (95% CI)	*P*	HR (95% CI)	*P*	HR (95% CI)	*P*
Age (year)	0.989 (0.969–1.009)	0.277	0.990 (0.970–1.011)	0.344	0.982 (0.963–1.001)	0.068
Total radiotherapy time (day)	1.018 (1.006–1.030)	**0.003**	1.017 (1.005–1.029)	**0.004**	1.018 (1.006–1.029)	**0.002**
Age (year)						
< 60	1.000		1.000		1.000	
≥ 60	0.829 (0.621–1.108)	0.205	0.868 (0.651–1.156)	0.333	0.770 (0.581–1.019)	0.068
Gender						
Female	1.000		1.000		1.000	
Male	1.542 (1.071–2.220)	**0.020**	1.522 (1.062–2.181)	**0.022**	1.509 (1.062–2.144)	**0.022**
ECOG PS						
0–1	1.000		1.000		1.000	
2–3	0.897 (0.652–1.235)	0.507	0.936 (0.683–1.282)	0.681	0.900 (0.660–1.227)	0.506
Tumor length (cm)						
< 8	1.000		1.000		1.000	
≥ 8	1.307 (0.946–1.805)	0.105	1.355 (0.984–1.866)	0.063	1.332 (0.973–1.824)	0.074
Total radiotherapy time (day)						
< 49	1.000		1.000		1.000	
≥ 49	1.951 (1.398–2.722)	**< 0.001**	2.051 (1.473–2.855)	**< 0.001**	1.892 (1.362–2.627)	**< 0.001**
T stage						
1–2	1.000		1.000		1.000	
3–4	3.266 (1.818–5.870)	**< 0.001**	3.004 (1.708–5.284)	**< 0.001**	3.363 (1.913–5.911)	**< 0.001**
N stage						
0–1	1.000		1.000		1.000	
2–3	0.825 (0.586–1.161)	0.270	0.839 (0.598–1.177)	0.310	0.982 (0.711–1.357)	0.912
AJCC stage						

Variables	OS		RFS		DMFS	
	HR (95% CI)	*P*	HR (95% CI)	*P*	HR (95% CI)	*P*
I–II	1.000		1.000		1.000	
III–IV	1.566 (1.064–2.305)	**0.023**	1.538 (1.050–2.251)	**0.027**	1.741 (1.191–2.545)	**0.004**
IC cycles (times)						
0	1.000		1.000		1.000	
1/2	0.923 (0.645–1.321)	0.660	0.864 (0.604–1.235)	0.422	0.893 (0.630–1.267)	0.526
3/4	0.470 (0.220–1.004)	0.051	0.442 (0.207–0.945)	**0.035**	0.629 (0.321–1.233)	0.177

Hazard ratios and 95% confidence intervals were calculated by a stratified Cox proportional hazards model. *P* values less than 0.05 are highlighted in bold.

AJCC stage = American Joint Committee on Cancer stage; DMFS = distant metastasis-free survival; ECOG PS = Eastern Cooperative Oncology Group performance status; IC = induction chemotherapy

SUPPLEMENTARY TABLE 3. Multivariate Cox analysis of overall survival (OS), recurrence-free survival (RFS), and distant metastasis-free survival (DMFS) before propensity score matching (PSM)

Variables	OS		RFS		DMFS	
	HR (95%CI)	*P*	HR (95%CI)	*P*	HR (95%CI)	*P*
Gender						
Female	1.000		1.000		1.000	
Male	1.553 (1.079–2.236)	0.018	1.637 (1.139–2.351)	0.008	1.515 (1.066–2.153)	0.021
Total radiotherapy time (day)						
< 49	1.000		1.000		1.000	
≥ 49	1.780 (1.273–2.487)	0.001	1.860 (1.325–2.612)	< 0.001	1.713 (1.231–2.384)	0.001
T stage						
1–2	1.000		1.000		1.000	
3–4	3.006 (1.667–5.419)	<0.001	2.825 (1.598–4.994)	< 0.001	3.091 (1.753–5.452)	< 0.001
AJCC stage						
I– II						
III–IV						
IC cycles (times)						
0			1.000			
1/2			0.938 (0.652–1.352)	0.733		
3/4			0.373 (0.174–0.800)	0.011		

Hazard ratios and 95% confidence intervals were calculated by a stratified Cox proportional hazards model.

P values less than 0.05 are highlighted in bold.

SUPPLEMENTARY TABLE 4. Response to induction chemotherapy for different regimens

Indution chemotherapy regimens	Response		Total	*P**
	CR + PR n (%)	SD + PD n (%)		
PF	1 (12.5)	7 (87.5)	8	0.315 (PF vs.TP)
TP	10 (40.0)	15 (60.0)	25	0.020 (PF vs.TPF)
TPF	22 (57.9)	16 (42.1)	38	0.165 (TP vs.TPF)

*A *P*-value of 0.017 or less after the Bonferroni correction was considered significant.

CR = complete response; PD = progressive disease; PR = partial response; PD = stable disease

PF = cisplatin 50 mg/m2/day on days 1 to 2 and 5-fluorouracil 500 mg/m2/day on days 1 to 3;

TP = docetaxel 60 mg/m2/day on day 1 or paclitaxel 150 mg/m2/day on day 1 and day 8, cisplatin 50 mg/m2/day on days 1 to 2);

TPF = docetaxel 60 mg/m2/day on day 1, cisplatin 50 mg/m2/day on days 1 to 2, and 5-fluorouracil 500 mg/m2/day on days 1 to 3

SUPPLEMENTARY TABLE 5. Adverse events during induction chemotherapy

Adverse event (n = 71)	Any grade n (%)	Grade 1-2 n (%)	Grade 3 n (%)	Grade 4 n (%)
Leucopenia	32 (45.1)	23 (32.4)	7 (9.9)	2 (2.8)
Neutropenia	32 (45.1)	23 (32.4)	7 (9.9)	2 (2.8)
Febrile neutropenia	3 (4.2)	2 (2.8)	1 (1.4)	0 (0)
Anemia	21 (29.6)	20 (28.2)	1 (1.4)	0 (0)
Thrombocytopenia	15 (21.1)	15 (21.1)	0 (0)	0 (0)
